# The Association of Digit Ratio (2D : 4D) with Cancer: A Systematic Review and Meta-Analysis

**DOI:** 10.1155/2018/7698193

**Published:** 2018-02-08

**Authors:** Adomas Bunevicius

**Affiliations:** Neuroscience Institute, Lithuanian University of Health Sciences, Kaunas, Lithuania

## Abstract

**Objective:**

Intrauterine sex hormone environment as indicated by the second to the fourth digit ratio (2D : 4D) can be associated with cancer risk. This systematic review and meta-analysis aimed to evaluate the association of 2D : 4D with cancer diagnosis, malignancy, and age at presentation.

**Methods:**

Studies that evaluated the association of 2D : 4D with cancer risk were collected from Pubmed/MEDLINE and Clarivate Analytics databases. Nineteen studies were included in the qualitative analysis.

**Results:**

The 2D : 4D ratio was studied in prostate cancer, breast cancer, testicular cancer, gastric cancer, oral cancer, brain tumors, and cervical intraepithelial neoplasia. Low 2D : 4D was associated with prostate cancer, gastric cancer, and brain tumors, while high 2D : 4D, with breast cancer risk and cervical dysplasia. The 2D : 4D ratio was not associated with prostate, breast, and gastric cancer stage. Greater 2D : 4D ratio was associated with younger presentation of breast cancer and brain tumors. The meta-analyses demonstrated that testicular cancer was not associated with right-hand 2D : 4D ratio (*p* = 0.74) and gastric cancer was not associated with right-hand (*p* = 0.15) and left-hand (*p* = 0.95) 2D : 4D ratio.

**Conclusions:**

Sex hormone environment during early development is associated with cancer risk later in life. Further studies exploring the link between intrauterine hormone environment and cancer risk are encouraged.

## 1. Introduction

Cancer remains among the leading causes of death and disability worldwide [1]. Global burden of cancer will continue to grow due to increasing age of expanding population [2]. Early detection of cancer is critical for optimized treatment outcomes and patient prognosis. Therefore, early identification of individuals at elevated cancer risk will become increasingly important for guiding targeted screening interventions and early diagnosis [3].

Epidemiologic, clinical, and experimental research findings strongly suggest that reproductive hormones have an important role in oncogenesis and progression of breast cancer, prostate cancer, and testicular cancer [4, 5]. There is an increasing recognition that events and environment (including hormones) during *in utero* period when organs are still developing can increase cancer risk later in life [6, 7]. The ratio of the lengths of the index (2D) and ring (4D) fingers (2D : 4D ratio) is a proposed biomarker for prenatal sex hormone (testosterone and estrogen) exposure [8]. Sex steroids modulate digit development during a narrow developmental window via androgen and estrogen receptors which are expressed in fetal cartilaginous tissues [9]. The 2D : 4D ratio is established during *in utero* period and is a stable signature of prenatal sex steroid exposure throughout life. Lower 2D : 4D ratio is associated with greater prenatal testosterone and lower estrogen exposure, and it has tendency to be higher in women [10]. Interest and number of research studies using the 2D : 4D ratio as a proxy of disrupted endocrine signaling during early development stages and its health implications are constantly rising [11]. Available evidence suggests that the 2D : 4D ratio is associated with behavioral, developmental, and somatic disorders and can have important health implications [12, 13]. It was hypothesized that the 2D : 4D ratio may be of value in the diagnosis and prognosis of sex-dependent cancers. However, to date, there are no studies, systematic reviews, and meta-analyses of the available research linking the 2D : 4D ratio with the risk to develop cancer and with cancer malignancy [14]. Identification of 2D : 4D as cancer risk factor could potentially contribute towards earlier cancer diagnosis via targeted screening interventions of individuals who are at elevated cancer risk.

This systematic review and meta-analysis aim to evaluate the association of 2D : 4D ratio with cancer diagnosis and tumor malignancy.

## 2. Methods

The review was implemented in accordance with the preferred reporting items for systematic reviews and meta-analyses (PRISMA) statement, and meta-analysis was performed according to the MOOSE group guidelines of observational meta-analyses [15].

### 2.1. Data Sources and Search Strategy

The systematic review was performed on July 16, 2017, to identify all available published studies that evaluated the association of the 2D : 4D ratio with cancer risk and cancer malignancy. Articles were identified from the Pubmed/MEDLINE and Clarivate Analytics databases using relevant keywords (mesh vocabulary and free text terms): “digit ratio,” “2D : 4D” and “cancer,” “tumor.” There were no restrictions to country of origin and publication date. Only original research papers performed in humans and with their abstracts or full texts available in English were considered for the review. Review papers, case reports, commentaries, editorials, and meeting abstracts were not included in the analysis. References of identified papers were reviewed for other relevant papers. Authors of unpublished data were not contacted.

### 2.2. Study Selection and Data Extraction

Initial literature analysis was performed by reviewing titles and abstracts of identified papers. Case-control and prospective cohort studies were included in the analyses if they (1) compared the 2D : 4D ratio in cancer patients versus control subjects, (2) investigated the association of 2D : 4D ratio with cancer malignancy and/or age at presentation, or (3) evaluated long-term cancer risk as a function of 2D : 4D ratio.

Relevant articles were extracted and subjected to full-text analyses. Selected articles were reviewed, and the following variables were extracted from the full text and/or abstracts of each paper: year and country of publication, cancer type, presence of control subjects, number of patients and controls studied, association of 2D : 4D ratio, and Dr-l with cancer and cancer malignancy/grade. The methodological quality of studies was evaluated according to the Newcastle-Ottawa Scale checklist for observational studies [16].

### 2.3. Statistical Analysis

Revman 5.3 software (The Cochrane Collaboration, Oxford, UK) was used for meta-analyses. Only cross-sectional studies that compared 2D : 4D ratios of both or one hand in cancer patients versus control subjects were included in the meta-analysis. Meta-analyses were performed separately for each cancer type and each hand (right and left). Only cancer types with more than one independent study investigating the association between 2D : 4D ratio and cancer were considered for meta-analyses. Analyses were performed separately for each cancer type. Data is presented using standard mean difference (SMD) and its 95% CI. Heterogeneity was evaluated by the *I*^2^ statistic.

## 3. Results

### 3.1. Qualitative Review

**Figure 1:**
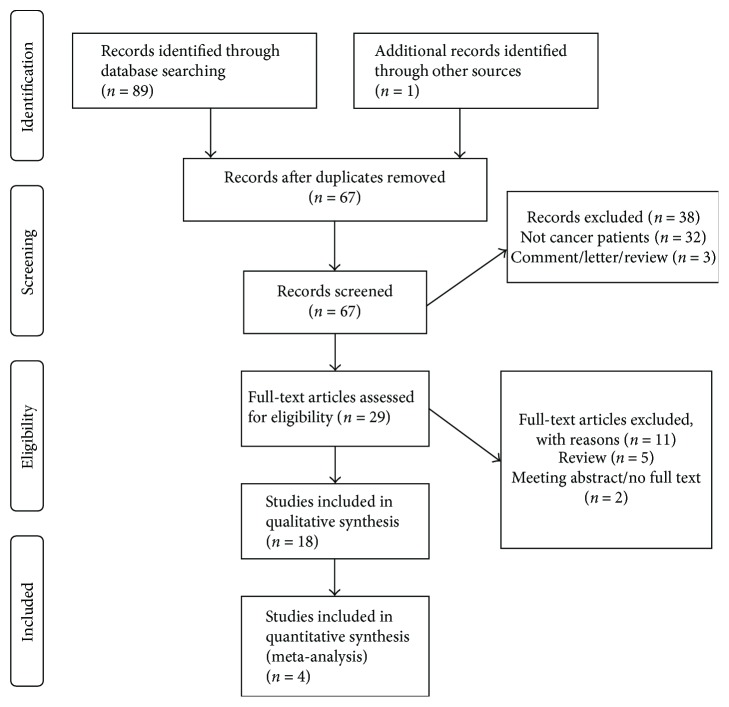
Flow chart of study selection.

Sixty-seven articles were identified during literature search (Figure 1). Screening of titles and abstracts results in exclusion of 38 studies, and 29 articles were selected for full-text review. Subsequently, 11 studies were excluded and 18 studies were included in the qualitative analysis (Table 1). Papers of the selected studies were published between 2010 and 2016. The number of cases in case-control and cross-sectional studies ranged from 25 [17] to 1524 [18] patients. The 2D : 4D ratio was studied in patients with prostate cancer (9 studies), breast cancer (2 studies), testicular cancer (2 studies), gastric cancer (2 studies), oral cancer (1 study), brain tumors (1 study), and cervical dysplasia (1 study).

### 3.2. Prostate Cancer

The 2D : 4D ratio has received the most attention in prostate cancer patients with a total of 8 case-control or cross-sectional studies (*n* = 4128 patients) and one prospective cohort study (*n* = 6458 men from community sample) [19] (Table 1). The majority of selected studies reported that prostate cancer was associated with lower 2D : 4D ratio [18, 20–22], while two groups did not find an association of 2D : 4D ratio with prostate cancer [23] or reported that greater 2D : 4D ratio was associated with prostate cancer [24].

A case-control study in 100 prostate cancer patients and 100 control subjects found that 2D : 4D ratio was lower in prostate cancer patients when compared to healthy controls [20]. Similarly, a study from UK that included 1524 prostate cancer cases and 3044 controls found that prostate cancer patients were less likely to select pictures with index finger longer than ring finger (high 2D : 4D ratio) from a series of pictures provided via postal survey [18]. Two studies from Korea that prospectively included a total of 1136 men aged 40 or older presenting with a PSA level of ≤40 ng/ml [22] and with lower urinary tract symptoms [21] found that 2D : 4D ratio of <0.95 was associated with greater prostate cancer detection rate. Findings from the prospective Melbourne Collaborative Cohort Study with a median of 16 years of follow-up suggested that higher 2D : 4D might be associated with decreased prostate cancer risk before 60 years of age [19]. On the other hand, a smaller study from Spain in 220 patient referred for transrectal biopsy for suspected prostate cancer reported that 2D : 4D of >0.95 was related to prostate neoplasia [24]. A study from Brazil in 474 men older than 40 years of age found that 2D : 4D ratios were similar between prostate cancer patients and individuals at high and at low risk for prostate cancer [23].

The majority of studies did not find an association of 2D : 4D ratio with Gleason score, metastatic status, and family history of prostate cancer [20, 25, 26] with the exception of one study that found inverse relationship of 2D : 4D ratio with core cancer volume and biopsy cores with high Gleason score in a study of 408 positive biopsy cores [21].

### 3.3. Breast Cancer

Two studies reported that the presence of breast cancer and earlier age at diagnosis were associated with greater 2D : 4D ratio. A study from China in 109 breast cancer patients and 109 controls found that right and left-hand 2D : 4D ratios were higher in breast cancer patients and correlated negatively with age at disease presentation [27]. The Melbourne Collaborative Cohort Study that followed 9044 women for a median of 16 years found that breast cancer risk was directly associated with left 2D : 4D ratio and inversely with Dr-l, but not with right 2D : 4D ratio [28]. Greater right 2D : 4D and Dr-l were also associated with younger age at breast cancer diagnosis.

### 3.4. Testicular Cancer

The association of 2D : 4D ratio with testicular germ cell tumors was examined in two studies with a combined sample size 317 patients [29, 30]. Both studies found that 2D : 4D ratio was similar between cancer patients and controls.

### 3.5. Gastric Cancer

Two studies addressed the potential association of 2D : 4D ratio with gastric cancer and reported opposite findings. A study from Brazil in 57 patients with gastric cancer and 59 controls found that right-hand 2D : 4D ratio was greater and Dr-l was lower in cancer patients [31]. On the other hand, a study from China found that right and left-hand 2D : 4D ratios were lower in 94 male patients with gastric cancer relative to 91 controls [32]. In both studies, the 2D : 4D ratio was not associated with cancer staging, tumor size (T), regional lymph node involvement (N), or distant metastases (M).

### 3.6. Oral Cancer

A study in 25 oral squamous cell carcinoma patients, 25 individuals with oral premalignant lesions, and 25 controls found that 2D : 4D ratio was greater in males with oral squamous cell carcinoma when compared to males with oral premalignant lesions and controls [17].

### 3.7. Brain Tumors

A study that included 85 patients with brain tumors, including meningioma, glioblastoma, pituitary adenoma, and low-grade glioma found that right and left-hand 2D : 4D ratios were lower in brain tumor patients than control subjects [33]. Greater left 2D : 4D ratio and lesser Dr-l were associated with younger age at presentation.

### 3.8. Cervical Intraepithelial Neoplasia

A study from the United Kingdom in 90 adolescents and 240 nonadolescents stratified by the human papillomavirus (HPV) status and presence of cervical intraepithelial neoplasia (CIN) found that women with any degree of cervical dysplasia were significantly more likely to have a higher 2D : 4D ratio when compared with women who were HPV-negative [34]. There was nonsignificant trend for the association of higher 2D : 4D ratio with persistent HPV infection. The 2D : 4D ratio was similar for HPV-positive and HPV-negative women with normal smears.

### 3.9. Meta-Analysis

**Figure 2:**
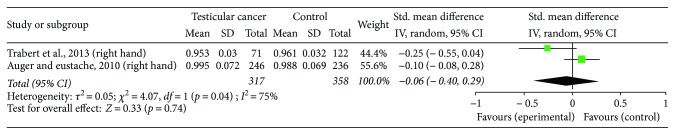
The association of the right-hand 2D : 4D ratio and testicular cancer.

**Figure 3:**
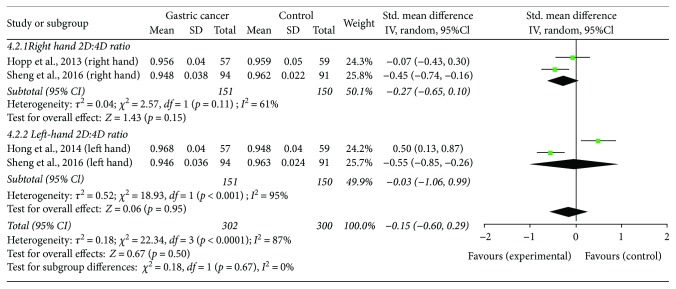
The association of the right-hand and left-hand 2D : 4D ratio with gastric cancer.

Four independent cross-sectional studies that compared right and/or left-hand 2D : 4D ratios in cancer patients versus control subjects were included in the meta-analysis. The studies eligible for the meta-analysis included patients with testicular cancer (two studies; 563 patient and 594 control hands; Figure 2) and gastric cancer (2 studies; 302 patient and 300 control hands; Figure 3) (Table 1). The study quality was adequate (Table 2). Due to high heterogeneity (*I*^2^ ≥ 61%), a random effect model was used. The meta-analyses demonstrated that right-hand 2D : 4D ratio was not different between testicular cancer patients and controls (*p* = 0.74; Figure 2). Right-hand 2D : 4D ratio and left-hand 2D : 4D ratio were also similar between gastric cancer patients and control individuals (*p* = 0.15 and *p* = 0.95, respectively; Figure 3).

## 4. Discussion

Systematic review and meta-analysis suggest that sex hormone uterine environment can encode susceptibility to develop certain cancers later in life. Specifically, low 2D : 4D was associated with prostate cancer and brain tumors, while high 2D : 4D was associated with breast cancer and cervical dysplasia. Testicular cancer, gastric cancer, and oral cancer were not associated with 2D : 4D. The 2D : 4D ratio was not associated with prostate cancer, breast cancer, and gastric cancer stage. Greater 2D : 4D ratio was associated with younger age of breast cancer and brain tumor diagnosis.

The present study suggests that high prenatal testosterone and low estrogen exposure (i.e., lower 2D : 4D ratio) are associated with prostate cancer and brain tumors risk, while low testosterone and high estrogen exposure (i.e., high 2D : 4D), with breast cancer and cervical dysplasia. Low right and left 2D : 4D ratio was associated with gastric cancer in Chinese men [32], while another study in men and women from Brazil found the opposite association, that is, left-hand 2D : 4D ratio was higher in gastric cancer patients relative to controls [31]. The meta-analysis of both studies showed no association between right/left 2D : 4D ratio with gastric cancer. These findings suggest that sex hormone environment during early developmental period can encode the risk to develop certain cancers later in life; therefore, biological mechanisms underlying the observed associations warrant further research. Digit development is regulated by activity of 19 skeletogenic genes with estrogen with testosterone regulating their expression in the opposite directions [9]. Importantly, some of these genes were also strongly implicated in the development and progression of cancer. Specifically, the *Wnt5* and *Sox2* genes were implicated in the digit development but also in oncogenesis of breast cancer, prostate cancer, gliomas, and gastric cancer [35, 36]. Furthermore, the activity of some of these genes that play a role in digit development and cancer is regulated by sex steroids also in extraskeletal tissues [9]. For example, sex steroids modulate the Wnt pathway gene expression during early development stages of prostate gland [37] and brain [38]. These observations suggest that balanced uterine environment of estrogen and testosterone concentrations can be important for balanced oncogenesis-related gene activity in extraskeletal tissues. Further studies exploring the association between *in utero* hormonal environment with cancer-related gene activity could potentially aid in identifying biological mechanisms predisposing to cancer development later in life.

In the majority of the reviewed studies, the digit ratio was not associated with indexes of breast cancer and prostate cancer malignancy. These findings suggest that while intranatal hormonal environment can trigger the development of certain cancers, once the cancer develops, its behavior is independent from early-stage hormonal environment. There were a few attempts to study the association of 2D : 4D ratio with prostate cancer treatment response and prognosis. A recent study in 382 prostate cancer patients receiving hormone therapy found that greater 2D : 4D ratio was associated with reduced risk of cancer progression and cancer-specific mortality [39]. Others also found that high 2D : 4D ratio was associated with better response to dutasteride treatment [40]. Furthermore, it is well-documented that 2D : 4D ratio is associated with behavioral and emotional problems that can impair prognosis of cancer patients. Towards this end, further studies exploring the association of 2D : 4D ratio with cancer prognosis are encouraged since hormonal signatures can potentially be used to improve prognostic accuracy and to guide treatment of cancer patients.

The association between 2D : 4D ratio and cancer risk suggests that in the future, this simple to use and reliable measure can potentially be used to identify patients at elevated risk for cancer. It remains to be seen if 2D : 4D ratio can have potential clinical implications for selecting patients for targeted screening interventions. It would be interesting to see if assessment of 2D : 4D ratio adds additional prognostic value above and beyond already established clinical and molecular cancer risk factors that are currently used for screening guidance.

Assessment methods of 2D : 4D ratio were different between studies and included caliper method [30], digit photograph analysis [33], self-reported measurement [29], and self-reporting using a series of pictures [18]. Studies examining the most reliable 2D : 4D assessment methods have shown that intraobserver and interobserver reliability was the greatest for computer-assisted techniques, followed by photocopies, physical measurements, and printed scans [41]. However, indirect 2D : 4D ratio measurement methods tend to distort 2D : 4D ratio downwards and this effect is sex dependent, such that it is the greatest in males relative to females (for detailed discussion on this issue, please see a recent review by Ribeiro et al. [42]). Further research studies advising the most optimal 2D : 4D measurement methods are encouraged.

The study has limitations. Firstly, meta-analyses were not performed for the majority of cancers due to lack of independent studies; thus, further studies in independent sample of patients aiming to replicate identified associations are encouraged. The number of studies and sample sizes were small of the studies included in the meta-analyses; therefore, meta-analyses were underpowered. Due to methodological heterogeneity across studies in terms of 2D : 4D ratio, measurement and outcome assessment qualitative analysis of included studies was performed. Also, although meta-analyses were performed separately for each cancer diagnosis, studies were heterogeneous in sample size, demographic characteristic, and age. Finally, results are at risk for publication bias because two studies that were presented in meeting abstracts were not identified.

## 5. Conclusions

Sex hormone environment during early development is associated with cancer risk later in life. High prenatal testosterone and low estrogen exposure (i.e., low 2D : 4D ratio) are associated with prostate cancer, gastric cancer, and brain tumors risk, while low testosterone and high estrogen exposure (i.e., high 2D : 4D), with breast cancer and cervical dysplasia. The 2D : 4D ratio is not associated with testicular cancer, breast cancer, and gastric cancer stage. Greater 2D : 4D ratio is associated with younger age of breast cancer and brain tumor diagnosis. Further studies exploring the association of 2D : 4D ratio in cancer prognosis and treatment response are encouraged.

## Figures and Tables

**Table 1:** Characteristics of the included studies.

	Authors/country	Country	Sample size (s)	Major findings
	*Prostate cancer*			
(1)	Mendes et al., 2016 [20]	Brazil	100 prostate cancer patients and 100 healthy individuals	Right and left 2D : 4D were lower in prostate cancer patients relative to controls Dr-l was not different between groups 2D : 4D did not correlate with Gleason score
(2)	Stolten et al., 2016 [26]	USA	452 prostate cancer patients	2D : 4D was not related to Gleason score, family history, or metastatic status
(3)	Garcia-Cruz et al., 2012 [24]	Spain	204 patients undergoing prostate biopsy	2D : 4D > 0.95 was related to neoplasia (OR = 4.4)
(4)	Waters et al., 2013 [25]	USA	238 prostate cancer patients	African-American men with prostate cancer were 3.70 times more likely to have low 2D : 4D than Caucasian men with prostate cancer 2D : 4D was not related to presence of metastasis, Gleason score, family history, or age at diagnosis
(5)	Oh et al., 2012 [21]	Korea	770 consecutive men aged 40 years or older that presented with lower urinary tract symptoms	Right 2D : 4D < 0.95 was associated with higher cancer detection rate and greater core cancer volume and higher Gleason score
(6)	Muller et al., 2011 [19]	Australia	6258 men from community sample	2D : 4D was not associated with prostate cancer risk in all participants There was a weak inverse association between 2D : 4D and risk of prostate cancer for age < 60
(7)	Rahman et al., 2011 [18]	UK	1524 prostate cancer cases and 3044 population-based controls	High right 2D : 4D was associated with lower prostate cancer risk
(8)	Jung et al., 2011 [22]	Korea	366 > 40 years old men with a PSA level ≤ 40 ng/ml presenting for lower urinary tract symptoms	Patients with 2D : 4D < 0.95 were more likely to have prostate cancer on biopsy
(9)	Salomao et al., 2014 [23]	Brazil	474 men > 40 years old: with prostate cancer (*n* = 222); high risk for prostate cancer (*n* = 82); and low risk for prostate cancer (*n* = 170)	2D : 4D was not different between the three groups

	*Breast cancer*			
(10)	Hong et al., 2014 [27]	China	109 breast cancer patients and 109 controls	Right and left 2D : 4D was higher in patients than controls Right and left 2D : 4D (but not Dr-l) correlated negatively with age at disease presentation
(11)	Muller et al., 2012 [28]	Australia	9044 women from community sample	Left 2D : 4D was positively and Dr-l was inversely associated with breast cancer risk Right 2D : 4D was not related to breast cancer risk Right 2D : 4D and Dr-l were inversely associated with age at breast cancer diagnosis

	*Testicular cancer*			
(12)	Trabert et al., 2013 [29]^A^	USA	246 testicular germ cell tumor patients and 236 controls	Right and left 2D : 4D and Dr-l were not associated with testicular germ cell tumor risk
(13)	Auger and Eustache, 2010 [30]^A^	France	71 testicular cancer patients and 122 controls	2D : 4D and Dr-l were not different between the two groups

	*Gastric cancer*			
(14)	Sheng et al., 2016 [32]^A^	China	94 male patients with gastric cancer and 91 controls	Right and left 2D : 4Ds were lower in gastric cancer patients than controls 2D : 4D ratio was not associated with tumor size, lymph node involvement or distant metastases, and age of onset
(15)	Hopp et al., 2013 [31]^A^	Brazil	57 patients with gastric cancer and 59 controls	Left 2D : 4D was higher and Dr-l was lower in patients relative to controls 2D : 4D did not correlate with cancer staging, tumor size, regional lymph node involvement, or distant metastases Low Dr-l was associated with adenocarcinomas

	*Oral cancer*			
(16)	Hopp and Jorge, 2011 [17]	Brazil	25 oral squamous cell carcinoma patients, 25 individuals with oral premalignant lesions, and 25 controls	2D : 4D was higher in males with oral squamous cell carcinoma than in males with oral premalignant lesions and controls

	*Brain tumor*			
(17)	Bunevicius et al., 2016 [33]	Lithuania	85 primary brain tumor (glioma, meningioma, and pituitary adenoma) patients and 106 controls	Right and left 2D : 4D were lower in brain tumor patients relative to controls In meningioma and glioma patients, age at presentation correlated negatively with left 2D : 4D and positively with Dr-l

	*Cervical intraepithelial neoplasia*			
(18)	Brabin et al., 2008 [34]	United Kingdom	Human papillomavirus (HPV) negative, normal smear (*n* = 120); HPV-positive, normal smear (*n* = 48); cervical intraepithelial neoplasia (CIN) 1 (*n* = 32); CIN 2/3 (*n* = 63)	2D : 4D ratio was similar for HPV-positive and HPV-negative women with normal smear Women with any degree of cervical dysplasia were significantly more likely to have a higher 2D : 4D when compared with HPV-negative women There was nonsignificant trend for the association of higher 2D : 4D ratio with persistent HPV infection

^A^Case-control studies included in the meta-analysis.

**Table 2:** Newcastle-Ottawa scale table of case-control studies included in meta-analysis.

	Selection	Comparability	Measurement	Total
Trabert et al., 2013 [29]	4	2	0	6
Auger and Eustache, 2010 [30]	4	0	1	5
Sheng et al., 2016 [32]	4	0	1	5
Hopp et al., 2013 [31]	4	2	1	7
